# Design of a school randomized trial for nudging students towards healthy diet and physical activity to prevent obesity: PAAPAS Nudge study protocol

**DOI:** 10.1097/MD.0000000000008898

**Published:** 2017-12-15

**Authors:** Diana Barbosa Cunha, Eliseu Verly Junior, Vitor Barreto Paravidino, Marina Campos Araújo, Mauro Felippe Felix Mediano, Michele Ribeiro Sgambato, Bárbara da Silva Nalin de Souza, Emanuele Souza Marques, Valéria Troncoso Baltar, Alessandra Silva Dias de Oliveira, Ana Carolina Feldenheimer da Silva, Federico J. Pérez-Cueto, Rosangela Alves Pereira, Rosely Sichieri

**Affiliations:** a Department of Epidemiology, Social Medicine Institute, State University of Rio de Janeiro; b Department of Physical Education and Sports, Naval Academy, Brazilian Navy; c Department of Epidemiology and Quantitative Methods, Sergio Arouca National School of Public Health, Oswaldo Cruz Foundation, Ministry of Health; d Evandro Chagas National Institute of Infectious Diseases, Oswaldo Cruz Foundation, Rio de Janeiro; e Department of Epidemiology and Biostatistics, Institute of Collective Health, Fluminense Federal University, Niterói; f Department of Social Nutrition, Epidemiology, Nutition Institute, State University of Rio de Janeiro, Rio de Janeiro, RJ, Brazil; g Department of Food Science, University of Copenhagen, Copenhagen, Denmark; h Department of Social and Applied Nutrition, Federal University of Rio de Janeiro, Rio de Janeiro, RJ, Brazil.

**Keywords:** adolescent, choice behavior, food consumption, obesity, prevention

## Abstract

**Objective:**

To evaluate the effectiveness of nudge activities at school on the students’ body mass index (BMI).

**Design:**

School-based factorial randomized community trial.

**Setting:**

Eighteen public schools in the municipality of Duque de Caxias, metropolitan area of Rio de Janeiro, Brazil.

**Participants and intervention:**

The 18 schools will be randomized into 4 group arms: group 1—control (without any activity); group 2—will receive educational activities in the classroom; group 3—will receive changes in the school environment (nudge strategies); group 4—will receive educational activities and changes in the school environment. Activities will occur during the 2018 school-year.

**Main outcome measure(s):**

The primary (BMI) and secondary (body fat percentage) outcomes will be assessed at baseline and after the study using a portable electronic scale with a segmental body composition monitor. The height will be measured by a portable stadiometer.

**Analysis:**

Statistical analyses for each outcome will be conducted through linear mixed models that took into account the missing data and cluster effect of the schools.

## 1 Introduction

In Brazil, 1 in 5 adolescents are overweight or obese.^[1]^ Environmental factors have great influence on the genesis of the obesity epidemic, due mainly to the consumption of energy-dense foods, low levels of physical activity, and sedentary lifestyle. Thus, these 2 components—diet and energy expenditure—are the cornerstones of prevention and treatment programs of obesity.^[2,3]^

Interventions in schools to prevent obesity have been established as an important strategy to address the problem, but previous research has shown that interventions towards healthy behaviors have had humble results.^[4]^ Observed effects imply high levels of knowledge, awareness, and positive attitude towards healthy eating,^[5]^ yet underscoring the problem that information and positive attitudes are not enough to produce actual change.

In this context, some interventions have used nudge strategy to alter people's behavior in a predictable way to make healthy behavior more likely.^[6,7]^ Nudging is an aspect of the choice architecture (the way of presentation and framing of choices) that alters the individuals’ choice in a predictable and desirable way, without forbidding any options or significantly changing their economic incentives,^[8,9]^ assuming that individual behavior is strongly influenced by the environment in which decisions are taken.^[10]^

Some studies conducted in developed countries have reported success in nudge interventions. Williamson et al^[11]^ observed positive results in food consumption after several changes in the school cafeteria environment. Wansink et al^[12]^ found a positive effect in making vegetables more attractive, by giving them nicknames, increasing their consumption in American school cafeterias. However, some studies did not find significant association between the use of nudge and healthier eating by the adolescents investigated. Bucher et al^[13]^ observed that point-of-purchase messages had no effect on vegetable basket sales in a college foodservice setting. Coyle et al^[14]^ showed that the presence of a salad bar was not associated with greater fruit and vegetable consumption in elementary schools.

Systematic reviews showed that the choice architecture seems effective to promote healthier food choices,^[13,15]^ and the most effective strategy was to increase food availability.^[16]^ However, the authors concluded that there is an important literature gap,^[7]^ and few high-quality studies evaluated nudge strategy in real-life scenarios^[13,15]^ and quantified the impact of these strategies on health outcomes, mainly obesity.^[13,17,18]^

Adolescents are highly susceptible to environmental influences, suggesting that nudging is an interesting tool in the prevention of obesity and school a strategy place to promote nudging.^[7]^

This project is a factorial trial which continues previous research called PAAPPAS study (Portuguese abbreviation of “parents, students, community health agents and teachers for healthy eating”).^[19]^ The present project will be conducted in schoolchildren, keeping the already consolidated components of primary prevention and including actions that aim to improve the adherence response to the interventions and providing a broad spectrum of actions to reach adolescence-based obesity prevention, including changes in School environment. More specifically, the present research aims to estimate the effect of “nudge” strategies on the students’ body mass index (BMI), from changes at the school environment, with the aim of creating school cafeterias that guide smarter food choices.

## 2 Methods

### 2.1 Study design and setting

PAAPPAS Nudge is a randomized community-based, school-based trial designed to promote healthful choices in a low-income scenario, which will be held in 2018 school-year in 18 municipal public schools in the municipality of Duque de Caxias, metropolitan area of Rio de Janeiro, Brazil (http://duquedecaxias.rj.gov.br/portal/). All students from the fifth and sixth grade from the 18 selected schools will be invited to participate to the study.

The study design will be of the factorial type, with 4 group arms:

1) Group 1: control (without any activity)
2) Group 2: will receive educational activities in the classroom
3) Group 3: will receive changes in the school environment (nudge strategies)
4) Group 4: will receive educational activities and changes in the school environment

In the factorial study, it is possible to verify both the main effect of each factor and an interaction of factors in the outcome variable.^[20]^

Schools will be sequentially numbered and randomized using opaque envelopes in the presence of investigators not involved in the current study. The statistical analysis will be conducted by investigators blinded to intervention allocation.

Participants in the Nudge control will receive individual encouragement.

The Municipality of Duque de Caxias is a poor area with a prevalence of overweight/obesity among adolescents of 24%.^[21]^

The study adhere to the Consolidated Standards of Reporting Trials (CONSORT) guidelines for cluster-randomized trials.^[22]^ Ethics approval for this study was obtained from Ethics Committee, and all the participants’ parents provided informed written consent.

### 2.2 Sample size calculation

Sample size of PAAPPAS Nudge was calculated based on a minimum effect of 0.15 in BMI variation and considering intra and intergroup comparisons. A sample size of 640 adolescents was required based on 95% power and a correlation between repeated measures of 0.5. Considering the intracluster correlation and refusal rate, the total sample size required was increased, leading 1300 adolescents, which can be achieved achieved with 10 schools with 4 to 5 classes of 30 students each one.

In addition, a subsample of 96 adolescents (48 boys and 48 girls) was calculated to detect a mean difference in daily energy expenditure of 110 kcal and a coefficient of variation equal to 1 (standard deviation [SD] of 110 kcal),^[23]^ with an α of 0.05 (2-sided) and β of 0.10.^[24]^ The adolescents were sampled from 1 intervention (n = 48) and 1 control school (n = 48).

### 2.3 Intervention

The activities will occur during the 2018 school-year and will combine different intervention strategies: educational interventions (in the classroom) and modifications in the environment (school canteens and sports court).

Group 2 will receive classroom-based educational activities based on an earlier study, which was developed in the same city and constituted a school-based intervention with the objective of reducing the consumption of sweetened beverages and biscuits, and increase the consumption of fruits, vegetables, and beans.^[25]^

In group 3, changes in the environment will be included that will facilitate both physical activity and healthy food choices. Interventions will be based on a nudge strategy, a tool of the architectural field of choice, which is defined as a way of influencing choices without limiting personal decisions or having to implement actions or interventions of greater cost and operational difficulty.^[26]^ The concept of nudging is based on the theory of the double process originated in the field of psychology, which involves the division of human cognition into 2 systems: the reflective and the automatic. The first is rational and involves conscious decisions, whereas the latter is more controlled by instinct.^[27,28]^ These 2 systems are taken into account for a number of cognitive biases, which explains, for example, why people, though conscious, have difficulty in translating intentions into actions. Using the understanding of these 2 biases is possible to changing behavior developing new ways of how food choices are presented in an environment, and individuals are unconsciously “motivated” to adopt healthy choices.

The approaches can be divided into passive and active. The passive approach, also called choice architecture, seeks to induce healthy choices through small changes in the environment. The active approach includes placing reminders in the environment. In the school context, the intention is to change the environment in which schoolchildren are inserted with the perspective of associating convenience with healthier practices and making less healthy practices less convenient, aiming to increase the consumption of healthier foods and stimulate the practice of physical activity. The convenience of healthy practices both preserves the power of choice (students are not required to consume healthy food or engage in physical activity) and encourages healthy behavior.^[17]^

Table 1 describes the intervention components and evaluation tools conducted in PAAPPAS Nudge.

**Table 1:**
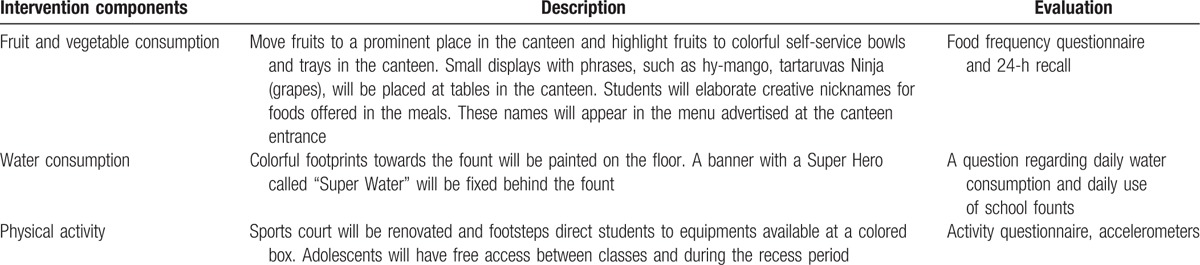
Intervention components, description, and evaluation.

Interventions regarding food consumption were based on those strategies of the Smarter Lunchrooms Movement (SLM)—an intervention study conducted in US school lunchrooms to led students to eat more fruits and vegetables, with positive results.^[29]^ In the present study, school lunchrooms will be the central place of modifications regarding food consumption, where the strategies that were effective in SLM will be reproduced:

1) Arrange the fruits served in the school lunch in a prominent position, in containers of bright and contrasting colors, to serve them. In this approach the student himself is served, which will probably result in his ingestion.
2) Creative names for food served at meals will be created by the students used on displays at canteen tables, posters on the way of the cafeteria, and at a menu outside the cafeteria. Increasing students’ expectancy increases their likelihood of choosing and eating new dishes. Students will be encouraged to participate actively in this stage. They will receive a list of the most commonly served vegetables at the lunch and will be asked to create fun-filled names for each of them, such as “carrots x-ray vision” and “super strength spinach.”

Moreover, to encourage water consumption, footprints that lead them to water fountains will be painted on the floor. To stimulate physical activity, several sports equipment (basketball hoops, volleyball nets, balls, ropes, shuttlecock, and a lot of toys) will be available for use in the school sports court. The courts will be renovated and footsteps will be painted to nudging students to the sports equipment.

Group 4 received both interventions.

The measurable intervention outcomes are fruits, vegetables, and water consumption (quantities in g or mL) obtained from the 24-hour recall, and estimated physical activity and daily energy expenditure.

### 2.4 Data collection

#### 2.4.1 Physical activity

Physical activity levels will be measured through a validated questionnaire including 6 questions about frequency and time of different physical activities domains (commuting to school, physical education classes, and other physical activities/sports) performed during the previous 7 days. Adolescents will be classified as active (≥300 minutes), insufficiently active (150–299 and 1–149 minutes), and inactive (no physical activity) according to their accumulated physical activity level.^[30]^ In addition, total energy expenditure will be measured using triaxial accelerometers (Actigraph, GT3X)^[31]^ in a subsample of 96 adolescents (48 boys and 48 girls). The accelerometers will be placed on the right hip (anterior iliac crest) during a 6-day period, except while bathing or during water activities.

#### 2.4.2 Food consumption

Food intake will be assessed by a food frequency questionnaire validated for teenagers in Rio de Janeiro^[32]^ and one 24-hour recall (Rec24-h). Netbooks containing a computer program developed for school use^[27,33]^ based on a technique called “Multiple Pass Method”^[34]^ will be used.

### 2.5 Expected outcomes

#### 2.5.1 Primary and secondary outcomes

The primary outcome of the study will be the variation of BMI and secondary outcome will be the variation of body fat percentage.

The body weight and body fat percentage will be measured by a portable electronic scale with a segmental body composition monitor (Tanita BC-558). The height will be measured by a portable stadiometer (brand: “AlturaExata”). The waist circumference will be measured with an inextensible measure tape, with amplitude of 150 cm and variation of 0.1 mm. All the measures will be taken according the protocol designed by Gordon et al.^[35]^ Each adolescent will be evaluated 3 times: baseline—at the begging of the school-year, the second—at the middle of the school-year; and the third—at the end of the school-year. To avoid follow-up losses, schools will be visited three times in each measurement time.

### 2.6 Statistical analysis

Statistical analyses for each outcome are conducted through linear mixed models that took into account the missing data and cluster effect of the schools. The models will be used to assess the impact of treatment (nudge or control school), time (treated as continuous, in months), and the treatment-by-time interaction. In this analysis, the distribution of probabilities more suitable for the variable of interest will be verified, and also the correlation structure between repeated measurements. Data analysis will be performed using the Statistical Analysis System, version 9.4 (SAS Institute Inc, Cary, NC).

### 2.7 Ethics and dissemination

The protocol was approved by the Ethics Committee of the Institute of Social Medicine (Comitê de ética do Instituto de Medicina Social—CAAE: 10471313.2.0000.5260). Written informed consent will be sent to all participants’ parents, by their children. All the researchers had access to the final trial dataset. The final dataset of the trial will be available for the researchers without names or any identification of the participants. The results of the study will be published in scientific journal and will be also sent to participants’ schools.

## 3 Discussion

Nudge strategies have been tested in developed countries with successful results in changing obesity-related behaviors.^[11,12,36]^ We are testing a combination of these strategies to improve adolescents’ eating and activity behaviors in schools of a low-income scenario.

In Brazil, the National School Food Program stipulates that all public schools should offer no cost daily meals for students, including fruits and vegetables on menus.^[37]^ However, data from a previous research carried out in the same locality of the present study^[25]^ indicate that a percentage of more than 30% of students do not consume the food offered at school.

Proving these low-cost nudge strategies as an effective step in changing obesity-related behaviors is an important and feasible tool to improve the consumption of school meals, in public schools in Brazil. Moreover, the inclusion of these strategies on a well-established school-based randomized trial will fill the gap in the literature to evaluate the nudge strategy in high-quality studies using real scenarios. The main study is now in progress, and the primary interventions started in July.

To the best of our knowledge, there are no studies on school nudge in Brazil. Moreover, nudge strategies to increase physical activity among children and adolescents have not been studied. The randomized controlled trial of the PAAPPAS Nudge intervention described in this protocol paper will provide data on the effects of choice architecture intervention approaches for increasing fruit and vegetable, and water consumption at the school environment among students in low-income countries, and also it is innovative in investigating the behavioral effects of environmental changes to encourage physical activity in a school setting.

## 4 Conclusions

The results of this study, if shown to be effective, will confirm the importance of incorporating environmental changes into traditional nutrition education interventions to facilitate healthier choices. Thus, the research will provide new tools to contribute to reduction of growing prevalence of obesity and poor nutrition in adolescents.
